# Identification of novel tumor microenvironment-associated genes in gastric cancer based on single-cell RNA-sequencing datasets

**DOI:** 10.3389/fgene.2022.896064

**Published:** 2022-08-15

**Authors:** Xujin Wei, Jie Liu, Zhijun Hong, Xin Chen, Kang Wang, Jianchun Cai

**Affiliations:** ^1^ The Graduate School of Fujian Medical University, Fuzhou, China; ^2^ Department of Gastrointestinal Surgery, Zhongshan Hospital of Xiamen University, Institute of Gastrointestinal Oncology, School of Medicine, Xiamen University, Xiamen, China; ^3^ Xiamen Municipal Key Laboratory of Gastrointestinal Oncology, Xiamen, China

**Keywords:** gastric cancer, single-cell RNA sequencing, tumor microenvironment, immune infiltration, prognostic biomarker

## Abstract

Tumor microenvironment and heterogeneity play vital roles in the development and progression of gastric cancer (GC). In the past decade, a considerable amount of single-cell RNA-sequencing (scRNA-seq) studies have been published in the fields of oncology and immunology, which improve our knowledge of the GC immune microenvironment. However, much uncertainty still exists about the relationship between the macroscopic and microscopic data in transcriptomics. In the current study, we made full use of scRNA-seq data from the Gene Expression Omnibus database (GSE134520) to identify 25 cell subsets, including 11 microenvironment-related cell types. The MIF signaling pathway network was obtained upon analysis of receptor–ligand pairs and cell–cell interactions. By comparing the gene expression in a wide variety of cells between intestinal metaplasia and early gastric cancer, we identified 64 differentially expressed genes annotated as immune response and cellular communication. Subsequently, we screened these genes for prognostic clinical value based on the patients’ follow-up data from The Cancer Genome Atlas. *TMPRSS15, VIM, APOA1,* and *RNASE1* were then selected for the construction of LASSO risk scores, and a nomogram model incorporating another five clinical risk factors was successfully created. The effectiveness of least absolute shrinkage and selection operator risk scores was validated using gene set enrichment analysis and levels of immune cell infiltration. These findings will drive the development of prognostic evaluations affected by the immune tumor microenvironment in GC.

## Introduction

Although the incidence and mortality rates have been declining worldwide, gastric cancer (GC) remains a common and lethal malignancy, especially in Asian countries (Smyth et al., 2020). In addition to traditional chemotherapy and surgery, adjuvant therapies, such as molecularly targeted therapy (Mundekkad and Cho, 2022) and immunotherapy, are gradually emerging as the staples of GC treatment. Uncovering the molecular mechanisms of the initiation and progression of GC is therefore critical for improving therapeutic efficacy. Molecular markers help deepen our understanding of GC subtypes (Bijlsma et al., 2017; Zhao et al., 2019), and with the technological advances in high-throughput sequencing, the focus on tumor heterogeneity is driving progress in precision medicine (Zeng and Jin, 2021) simultaneously. There is growing evidence that intra-tumoral heterogeneity includes not only genomic features but also the complex tumor microenvironment (TME). It is well recognized that TME comprises various stromal cells, abundant angiogenesis, and immune cell infiltration (Hanahan, 2022). Understanding the “soil” on which the “seed” grows into a tumor has essential implications for the diagnosis and treatment of GC.

Single-cell RNA sequencing (scRNA-seq) is a potent tool to obtain complete RNA transcripts at the level of single cells by RNA extraction, reverse transcription, amplification, and sequencing (Tang et al., 2009; Ramskold et al., 2012; Picelli et al., 2014). Compared with traditional sequencing methods of tumor tissue, scRNA-seq solves the problem that individual differences between cell types are ignored when the expression of all the genes in thousands of cells is averaged. It shows clear superiority in studying the diversity of tumor cell lineages and predicting interactions between cancer and the microenvironment (Muller and Diaz, 2017; Kumar et al., 2021). In terms of GC research, scRNA-seq shed light on the transcriptome network at different stages of the disease process, from atrophic gastritis, intestinal metaplasia (IM), and dysplasia, to early gastric cancer (EGC) (Zhang et al., 2019), as well as the spatial heterogeneity of microenvironment-related cells in diffuse-type GC (Jeong et al., 2021). Performing scRNA-seq of metastatic GC, the origins of transcriptomic heterogeneity in peritoneal carcinomatosis were analyzed (Wang R. et al., 2021), identifying *CLDN11* and *CDK12* as markers of lymph node metastasis (Wang B. et al., 2021). Nonetheless, combining large amounts of scRNA-seq data with multi-omics datasets and determining the clinical implications remains a challenge (Wang et al., 2022).

We data-mined existing single-cell transcriptome data from the Gene Expression Omnibus (GEO) to identify microenvironment-related cell types and draw signaling pathway networks according to the marker genes or intercellular communication-related genes. Enrichment analysis was performed to reveal the function of differentially expressed genes (DEGs) between IM and EGC. We leveraged clinical data from The Cancer Genome Atlas (TCGA) to establish a risk-scoring model by the LASSO-Cox regression algorithm. It contained the expression levels of *TMPRSS15, VIM, APOA1,* and *RNASE1,* which were highly correlated with clinical outcomes and immune cell infiltration. The results of our study may provide new options for prognostic biomarkers in GC.

## Materials and methods

### Data collection and pre-processing

ScRNA-seq data of normal cells and GC cells from the GSE134520 dataset were downloaded from the GEO database from the official website (https://www.ncbi.nlm.nih.gov/geo/) (Zhang et al., 2019), which had been pre-processed using CellRanger software. The dataset contains *Homo sapiens* samples sequenced by the HiSeq X Ten platform. A total of 13 biopsy specimens from the gastric antral mucosa of patients with non-atrophic gastritis, chronic atrophic gastritis, IM, and EGC were selected, and 56,440 cells were included in the dataset. Cells with an abnormal proportion of mitochondrial genes should be removed because it reflects the imbalance of cellular homeostasis and low cell quality. Given the potential existence of diploid cells, cells with genes < 200 or >5,800 were filtered out. Furthermore, the scDblFinder function in the Seurat R package was called to remove the double droplets. Finally, 48,566 cells were enrolled in our study.

The R package “TCGAbiolinks” (version 2.22.2) was used to obtain the gene expression in units of fragments per kilobase million (FPKM) for 407 patients with stomach adenocarcinoma (STAD) from TCGA. We also downloaded the clinical data for these patients, which include survival status (367 patients), stages, age, grades, and survival time. At the same time, the data of “Masked Somatic Mutation” and “Masked Copy Number Segment” called MuTect2 were downloaded.

### ScRNA-seq analysis using Seurat

First, we installed the R packages in R (version 4.1) and Seurat (version 4.0.5) (Satija et al., 2015), and used the merge function to merge the created Seurat object. To reduce the influence of different sequencing depths in cells, the normalization for raw counts was performed using the NormalizeData function (“LogNormalize” method), the top 2,000 variable features were identified using the Find Variable Genes function (“vst” method), and the data were integrated and scaled using ScaleData function. Subsequently, we performed principal component analysis (PCA) with the variable genes as inputs and identified the significant principal components whose *p*-value distributions were then visualized by the jackStraw function. In the FindNeighbors and FindClusters function with a resolution of 0.8, the Louvain algorithm was chosen for cell clustering. FindAllMarkers function with the Wilcoxon rank-sum test was carried out to identify specific marker genes, which compared expression values between cells in the cluster and all other cells. Finally, the results are represented with tSNE (t-distributed Stochastic Neighbor Embedding) dimension reduction by RunTSNE function.

### Cell annotation and identification of differentially expressed genes

Cell types were identified based on marker gene sets listed in Table 1. Subsequently, we used the FindAllMarkers function in Seurat to detect DEGs among the different cell types, using a *p*-value < 0.05 and |log2FoldChange (FC) | > 1 as the thresholds. Additionally, we used the FindAllMarkers function to analyze the differences between the EGC and IM groups to extract DEGs using the same thresholds previously stated. The intersection of three methods (“wilcox,” “t,” and “roc” test) was taken and transformed into a list of the final DEGs. Finally, the expression patterns of these DEGs in the different cell types were shown using heat maps, and the corrplot R package (version 0.92) was then used to perform a correlation analysis between these DEGs.

**TABLE 1:** Cell types of 25 clusters.

Cell type	Marker gene	Number of cell (total = 48,566 (%))
Gastric epithelial cells	*MUC6*, *TFF2*, *MUC5AC*, and *TFF1*	26,870 (55.33%)
Metaplastic stem-like cell	*OLFM4*, *EPHB2*, and *SOX9*	7570 (15.59%)
Enterocytes	*FABP1* and *APOA1*	5340 (11.00%)
T cell	*CD2* and *CD3D*	2574 (5.30%)
B cell	*CD79A*	2307 (4.75%)
Fibroblast	*DCN* and *PDPN*	1431 (2.95%)
Endothelial cell	*VWF* and *ENG*	1002 (2.06%)
Macrophage	*CSF1R* and *CD68*	479 (0.99%)
Mast cell	*TPSAB1*	384 (0.79%)
Goblet cell	*MUC2* and *ITLN1*	357 (0.74%)
Smooth muscle cell	*ACTA2*	252 (0.52%)

### Enrichment analysis of differentially expressed genes between early gastric cancer and IM groups

Gene Ontology (GO) enrichment analysis is commonly performed in large-scale functional enrichment analyses of genes on different dimensions and levels. It is generally performed on three levels, namely, biological process (BP), molecular function (MF), and cellular component (CC). Kyoto Encyclopedia of Genes and Genomes (KEGG) is a widely used database dealing with biological systems such as genomes, biological pathways, diseases, and drugs. The clusterProfiler (version 4.2.0) R package was used to perform GO functional annotation and KEGG pathway enrichment analysis for the DEGs between the EGC and IM groups to identify significantly enriched biological processes. The enrichment results were visualized using bar graphs and bubble graphs. An adjusted *p*-value of < 0.05 was defined as the significance threshold in the enrichment analysis.

### Analysis of cell-cell communication

The CellChat (http://www.cellchat.org/) R package (Jin et al., 2021) was used to calculate the intensity of cell–cell interactions and communication based on single-cell gene expression profiles and known ligands, receptors, and their cofactors. Significant ligand-receptor pairs were further identified based on the probability of receptor–ligand interactions and the results of perturbation testing. We then built a cell–cell communication network by adding the number or the intensity of significantly interacting ligand–receptor pairs between cell types. In addition, we visualized multiple ligand–receptor pairs or intercellular communications mediated by signaling pathways using bubble graphs to investigate the intensity of ligand–receptor interactions between cell types or the characteristics of ligands and receptors in terms of gene expression levels, including their commonalities or differences. Finally, we performed a systemic analysis of the cell–cell communication network by identifying the pathways that contributed most to the incoming and outgoing signals for specific cell groups, performing a network centrality analysis of the identified pathways, and then visualizing the network centrality scores.

### Mutation analysis

With the help of GenePattern (https://cloud.genepattern.org), the data of somatic mutations and copy number variations (CNVs) downloaded from TCGA database were analyzed by GISTIC 2.0 to assess the CNV events at the chromosomal arm level and the minimum common region between samples. Then, we use the maftools R package (Mayakonda et al., 2018) to visualize the aforementioned analysis results in the mutation annotation format, and the plotmafSummary function was used to plot the summary file.

### Construction of the prognostic model and clinical statistical analysis for model evaluation

To identify genes associated with the prognosis of gastric cancer, we selected the DEGs between the EGC and IM groups as candidate genes and performed univariate Cox regression analysis (*p* value < 0.01). Forest plots were created (R package: ggplot2) to display each variable’s *p*-value, hazard ratio (HR), and 95% confidence interval. We then performed least absolute shrinkage and selection operator (LASSO) regression analysis based on these prognostic genes to construct a prognostic model (R package: Glmnet, survival). The risk score was calculated by the formula:

$$Riskscore=\sum_{k=1}^{n}coef\left(k\right)*x\left(k\right).$$

Here, the *coef* (*k*) represents the LASSO-Cox regression coefficient, *n* represents the number of genes, and *x*(*k*) represents the expression value of each gene. The TCGA GC cohort was divided into low- and high-risk groups based on the median risk score, and then Kaplan–Meier analysis (R package: survival and survminer) was performed to analyze and compare the overall survival (OS) between the two subgroups. The time-dependent receiver operating characteristic (ROC) curve was adopted (R package: timeROC) to analyze the predictive accuracy and risk scores. Considering the clinical features, we also created a prognostic nomogram (R package: rms and survival) to predict the 1-, 3-, and 5-year overall survival.

### Gene set enrichment analysis

Gene set enrichment analysis (GSEA) is a computational method that determines whether a pre-defined set of genes shows statistically significant differences between two biological states and is usually performed to estimate the changes in pathways and biological process activities in gene expression dataset samples. To assess the differences in biological processes between the high- and low-risk groups based on the gene expression profiling datasets, we downloaded the reference gene sets (c2. cp.v7.2. symbols.gmt and c5. all.v7.2. symbols.gmt) from the MSigDB and used the GSEA method included in the clusterProfiler R package (Yu et al., 2012) to perform enrichment analysis of the datasets and visualize them. An adjusted *p*-value of <0.05 and a false discovery rate (FDR, q-value) < 0.25 were considered to suggest statistically significant differences.

### Estimation of immune cell-type fractions

The CIBERSORT (Chen et al., 2018) computational method was adopted to quantitatively convert the transcriptomic data of tumor tissues into the absolute abundance of immune cells and stromal cells, to evaluate the changes in the proportion of 22 human immune cell subpopulations. For each tumor sample, the sum of all the estimated immune cell-type fractions equaled 1.

## Results

### Analysis of 25 cell clusters from biopsy specimens of the human gastric antral mucosa with scRNA-seq data revealed high levels of cellular heterogeneity

**FIGURE 1:**
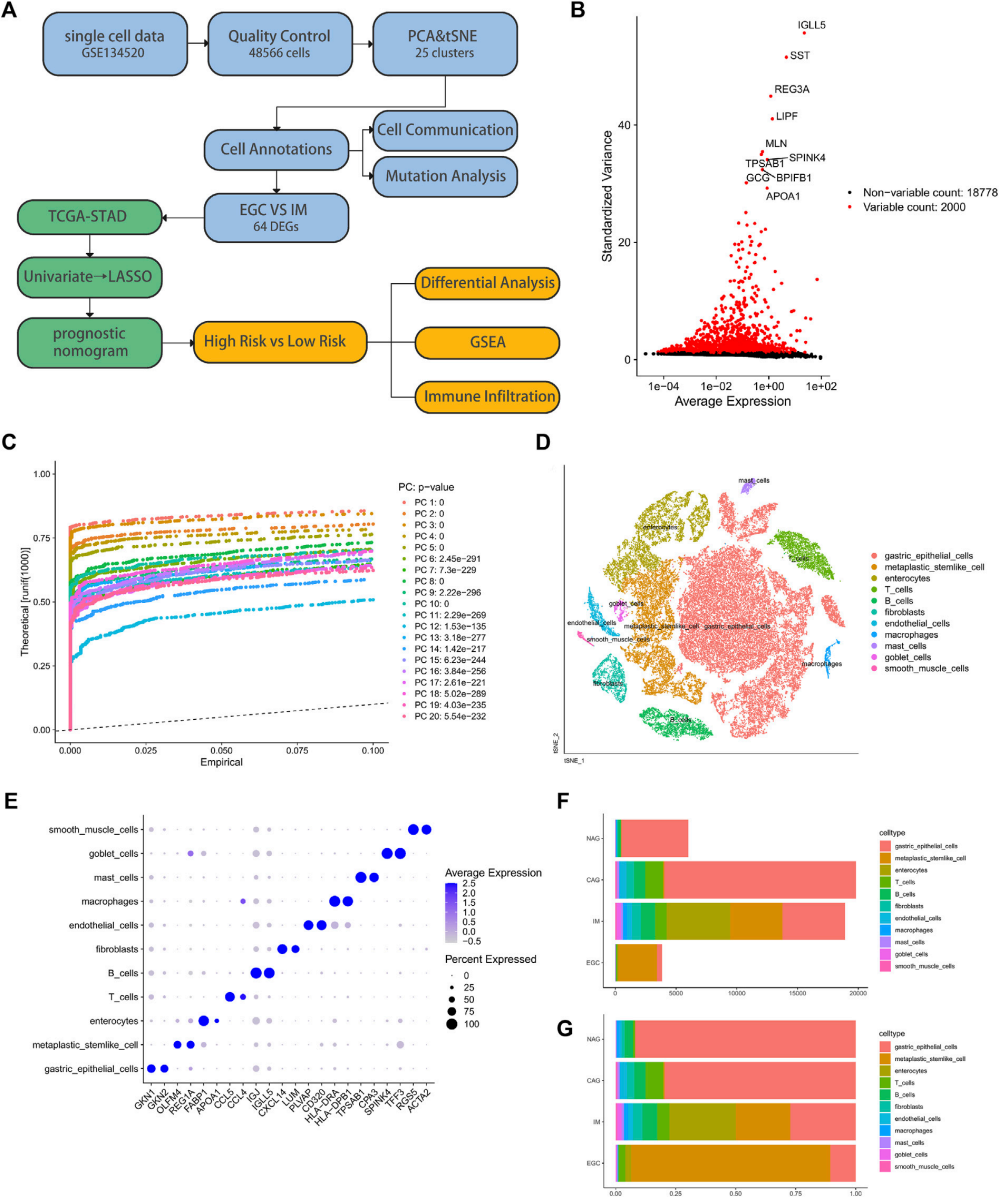
Analysis of the biopsy specimens from the gastric antral mucosa of patients with non-atrophic gastritis (NAG), chronic atrophic gastritis (CAG), intestinal metaplasia (IM), or early gastric cancer (EGC) based on single-cell RNA-seq data. **(A)** Flow chart of the study. **(B)** Scatter plot of standard deviation demonstrated the significantly differentially expressed genes between the cell types. **(C)** JackStrawPlot of 20 principal components (*p* value < 0.01) used to find clusters. **(D)** Cluster analysis based on the distribution of different cell types. **(E)** Prominent marker genes for each cell type. **(F)** and **(G)** Number of each cell type and the proportion of cells per cell type in the samples of the four different disease groups.

After filtration based on the quality control criteria and normalization of the scRNA-seq data, 48,566 cells were obtained (Supplementary Figure S1A). We selected 2,000 highly variable genes for downstream analysis and tagged the top ten (Figure 1B), such as *IGLL5*, *LIPF*, *TPSAB1*, and *APOA1*. PCA was performed to identify available dimensions and relevant genes, and 20 principal components (PC) were selected for subsequent analysis (Figure 1C). The tSNE algorithm was successfully applied to divide human cells of the gastric antral mucosa into 25 independent clusters (Figure 1D), which were identified by the marker genes for each cell type (Supplementary Figures S1B,C). Table 1 and Supplementary Figure S1D showed the numbers and percentages of each cell type identified with the 25 clusters. Specifically, gastric epithelial, metaplastic stem-like, and enterocytes accounted for 55.53%, 15.59%, and 11%, of the total cell count, respectively. We then identified DEGs between cell types (Supplementary Figure S1E) and used the top two genes with the most significant differential expressions to draw dot plots (Figure 1E). We also calculated the number of each cell type and the proportion of cells per cell type in the samples of the different disease groups (Figures 1F,G). The aforementioned results portray the diverse landscape of the microenvironment between tumor and non-tumor samples.

### Intercellular communication displayed locoregional immunomodulation in the carcinogenic process

**FIGURE 2:**
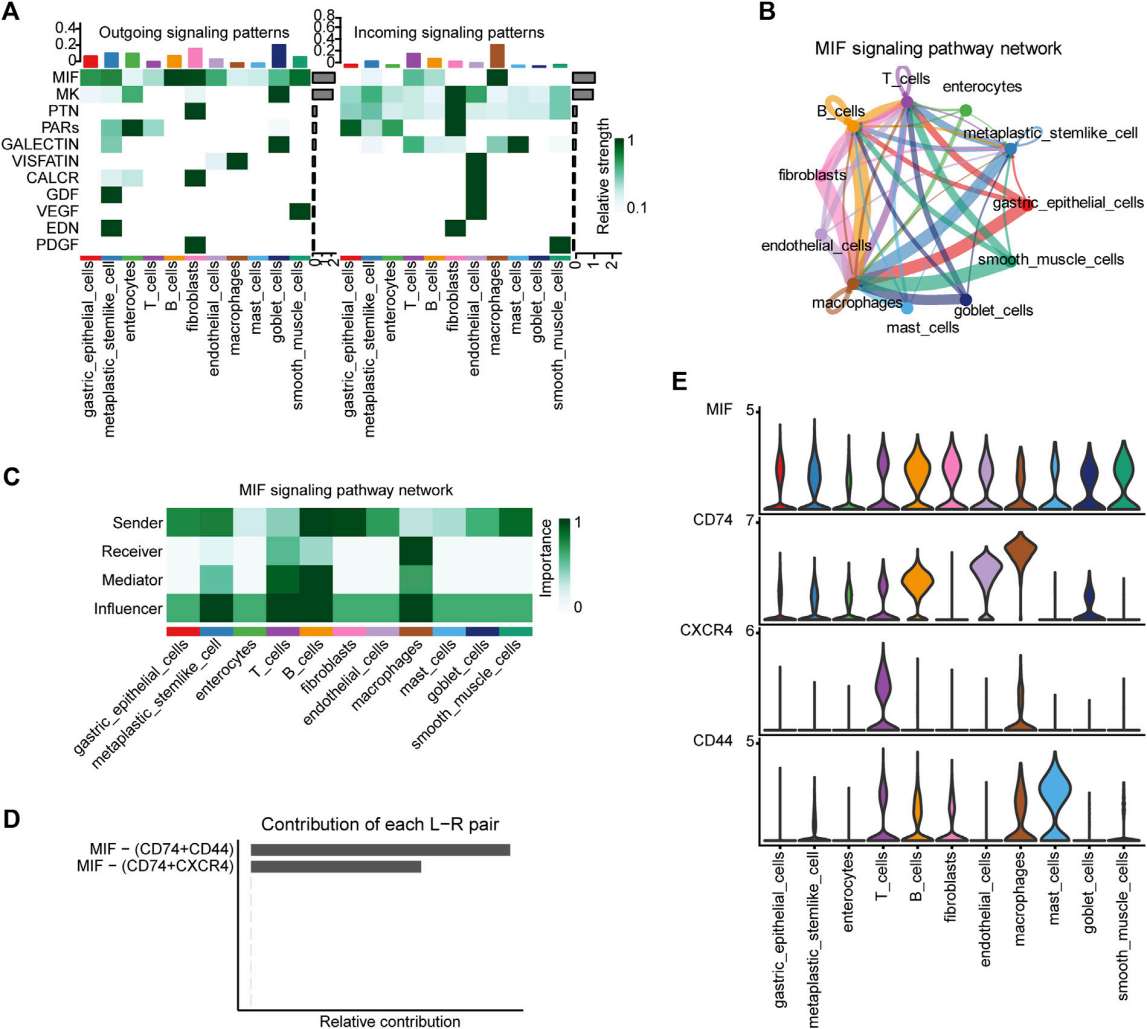
CellChat analyses of the intercellular communication network for the different cell types. **(A)** Contribution of the 11 pathways identified to the outgoing (left) and incoming (right) signals among the different cell types. **(B)** Intensity of cell–cell interactions in the MIF signaling pathway. **(C)** Network centrality scores of the MIF signaling pathway for each cell type. **(D)** Relative contribution of each ligand–receptor pair included in the MIF signaling pathway and **(E)** their expression patterns in the different cell types.

We detected a total of 11 signaling pathways in the 11 cell types annotated in the single-cell data using CellChat, including MIF, MK, PTN, PARs, and GALECTIN. Heat maps were generated to illustrate the contribution of each pathway to the incoming or outgoing signals among the cell types (Figure 2A, Supplementary Figure S2). We selected the MIF signaling pathway as it contributed the most amongst the cell types, used a circle plot to illustrate the intensity of cell–cell interactions (Figure 2B) and performed a network centrality analysis (Figure 2C) for this pathway. Clearly, the macrophage is the most dominant recipient; B cell, T cell, and metaplastic stem-like cell perform key roles in the network. Moreover, we demonstrated the expression patterns of all ligand-receptor pairs included in the MIF signaling pathway (Figure 2D,E; Supplementary Figure S3) in different cell types: CD74 is mainly expressed in macrophages, endothelial cells, and B cells; CD44 is predominantly expressed in mast cells; and CXCR4 is primarily expressed in T cells.

### Identification and enrichment analyses of differentially expressed genes between the early gastric cancer and IM groups

**FIGURE 3:**
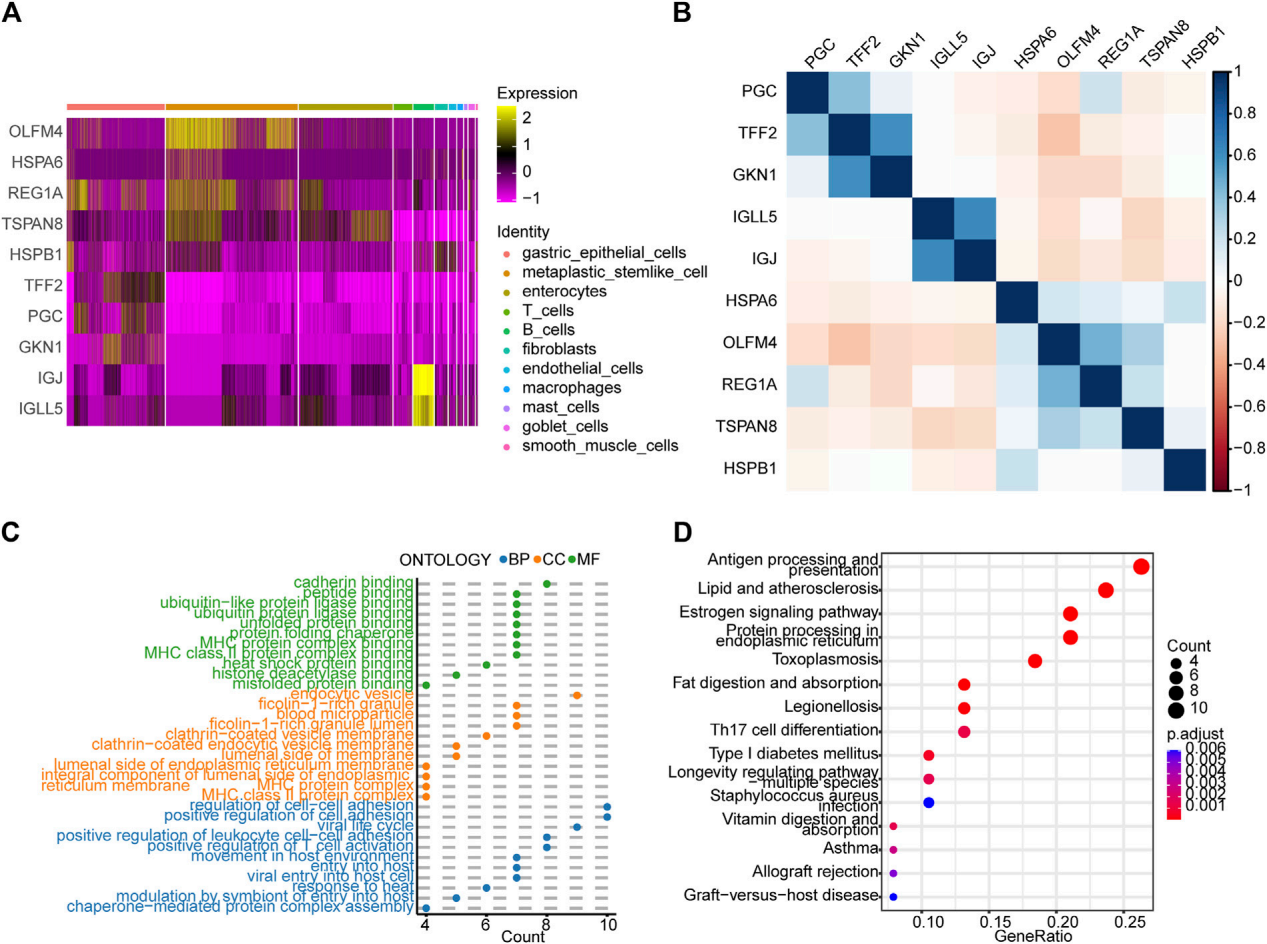
Analysis of differences between the early gastric cancer (EGC) and intestinal metaplasia (IM) groups and subsequent enrichment analyses. **(A)** Heat map showing the expression patterns of ten differentially expressed genes (DEGs) between the EGC and IM groups in the different cell types. **(B)** Heat map of the correlation between the DEGs. Dot plots showing the **(C)** GO and **(D)** KEGG pathway enrichment analyses of the DEGs (*p*-value < 0.01). BP, biological process; CC, cellular component; MF, molecular function.

By analyzing the differences between the EGC and IM groups, we identified 64 DEGs in total (*p*-value < 0.05 and |log2FoldChange| > 1). Heat maps (Supplementary Figure S4) were drawn to visualize the DEGs, and a correlation analysis of these genes was performed (Supplementary Figure S5). As shown in Figures 3A,B, *GKN1* and *TFF2* are highly correlated in gastric epithelial cells; *IGJ* and *IGLL5* are highly correlated in B cells; and *OLFM4, REG1A*, and *TSPAN8* are highly correlated in gastric epithelial cells. GO and KEGG pathway enrichment analyses of the DEGs (adj. *p* value < 0.05) were visualized using dot plots (Figures 3C,D). These results prove that cell communication pathways (cadherin binding, adj. *p* = 2.5 × 10^−4^; ficolin−1−rich granule, adj. *p*= 8.6 × 10^−6^; regulation of cell−cell adhesion, adj. *p* = 7.0 × 10^−5^; and estrogen signaling pathways) and inflammatory response pathways (MHC protein complex, adj. *p*= 1.6 × 10^−9^; endocytic vesicle, adj. *p* = 2.6 × 10^−5^; antigen processing and presentation, and Th17 cell differentiation) were enriched.

### Incidence of somatic mutations and copy number variations in 64 differentially expressed genes

**FIGURE 4:**
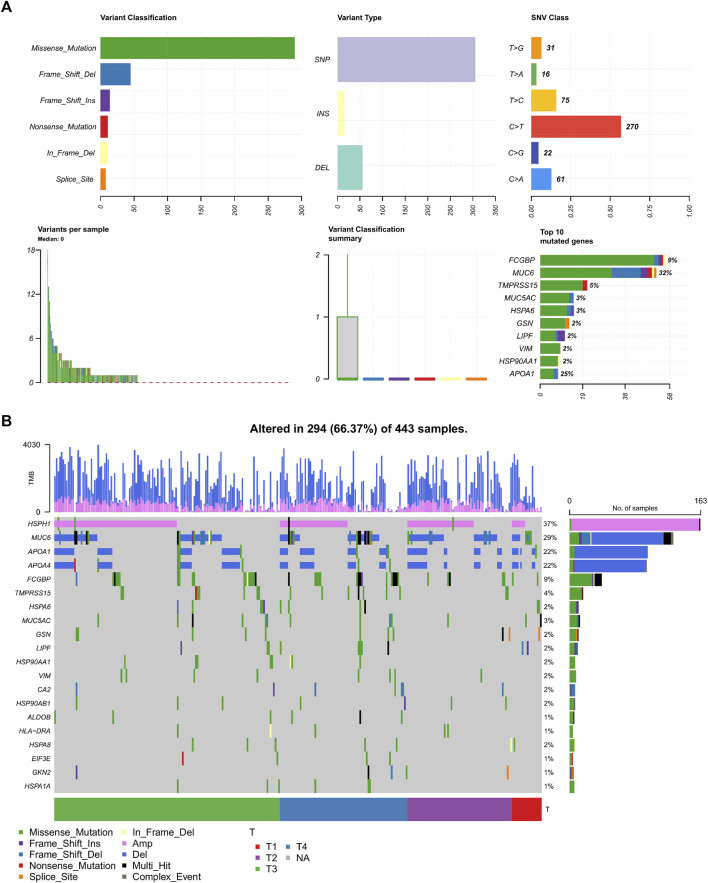
Mutational landscape in 64 DEGs. **(A)** Overview of the mutational frequency. **(B)** Mutation waterfall plot in the different T stages. SNP, single-nucleotide polymorphism; DEL, deletion; INS, insertion; SNV, single-nucleotide variant; Amp, amplification.

We next examined the mutational landscape of 64 DEGs in TCGA patients with STAD. We found that the missense mutations have the highest mutation rate and the single-nucleotide polymorphism (SNP) is the most occurred variant type (Figure 4A). Among these SNPs, the highest proportion of mutations occurred in the C to T transition mutations. *FCGBP* and *MUC6* are dominant in the top 10 mutated genes, and *TMPRSS15*, *VIM,* and *APOA1* are also included. Figure 4B showed that the *HSPH1* amplification (37%) and *MUC6* (29%) and *APOA1* (22%) frameshift deletions are the most common alterations observed. The mutagenesis in these TME-related DEGs is illustrated as the pro-carcinogenic potential.

### Construction of a TME-related prognostic model based on prognostic genes

**FIGURE 5:**
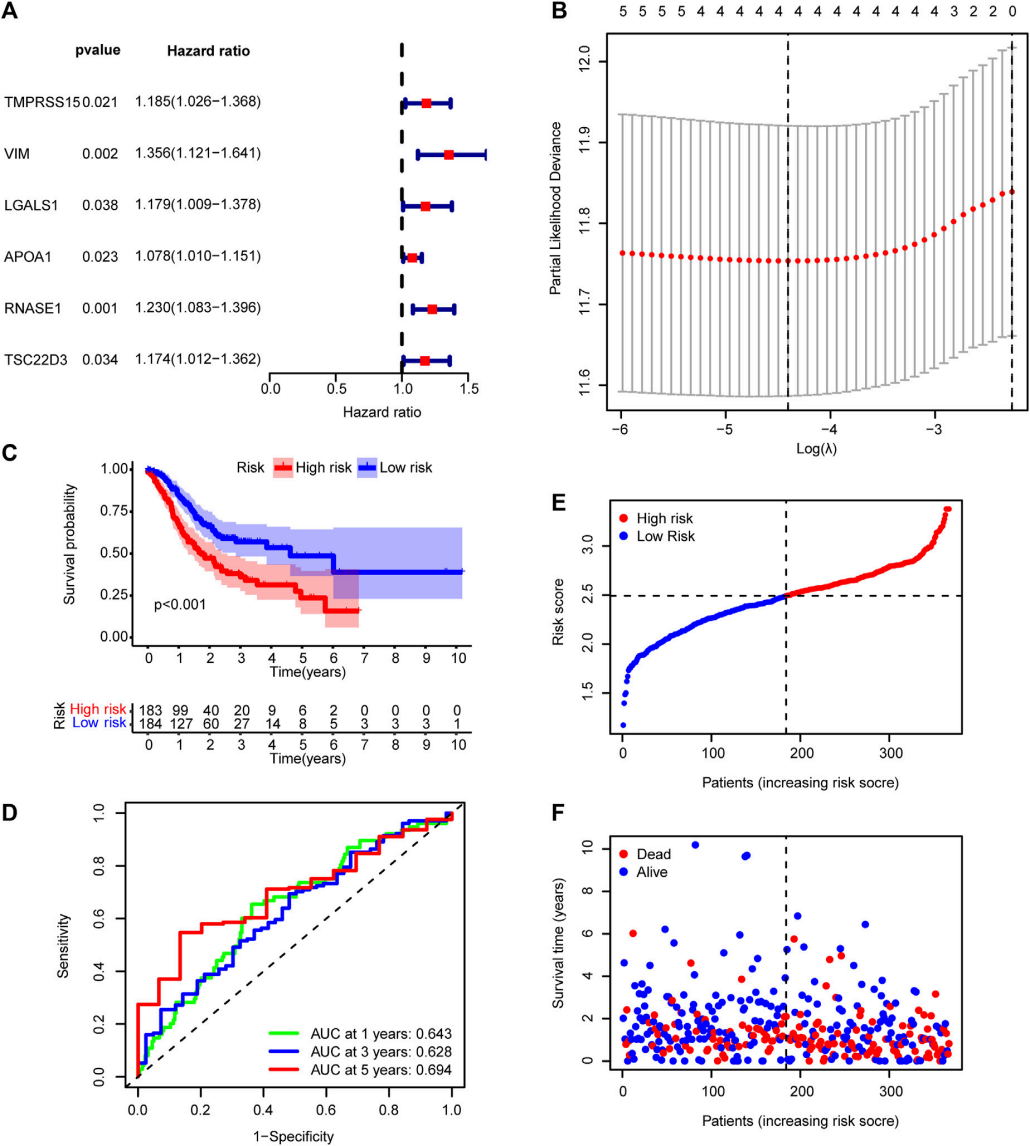
Prognostic model based on differentially expressed genes between the early gastric cancer and intestinal metaplasia groups. **(A)** Univariate Cox regression analysis showing the correlation between six genes and disease prognosis. **(B)** Diagram of error rates by 10-fold cross-validation. **(C)** Kaplan–Meier survival analysis for the high- and low-risk groups in TCGA cohort. **(D)** Time-dependent ROC curves showing the predictive accuracy of the prognostic model in TCGA cohort. **(E)** Risk score distribution and **(F)** survival status for patients in the TCGA dataset. AUC, area under the curve.

To evaluate the correlation between the prognosis for patients with GC and the expression of the 64 DEGs, we used univariate Cox regression analysis. The results suggested that six of the genes (*TMPRSS15*, *VIM*, *LGALS1*, *APOA1*, *RNASE1*, and *TSC22D3*) are significantly correlated with the disease prognosis (*p* < 0.05) (Figure 5A). In addition, the LASSO-Cox regression algorithm was used to establish a prognostic model, and the results suggested that four genes (*TMPRSS15*, *VIM*, *APOA1*, and *RNASE1*) are highly correlated with disease prognosis (Figure 5B). Risk score = (*TMPRSS15* ^∗^ 0.076) + (*VIM* ^∗^ 0.225) + (*APOA1* ^∗^ 0.066) + (*RNASE1* ^∗^ 0.135). The Kaplan–Meier plotter is an online tool to find survival biomarkers in GC based on the meta-analysis of GEO, TCGA, and European Genome-phenome Archive databases (Szász et al., 2016). Using the Kaplan–Meier plotter that has 875 enrolled patients with GC (Supplementary Figure S6), we further validated that these four genes have diagnostic value in association with adverse outcomes. We also verified their protein expression in the Human Protein Atlas database (Supplementary Figure S7), which is corroborated by the transcript levels in Supplementary Figure S4.

**FIGURE 6:**
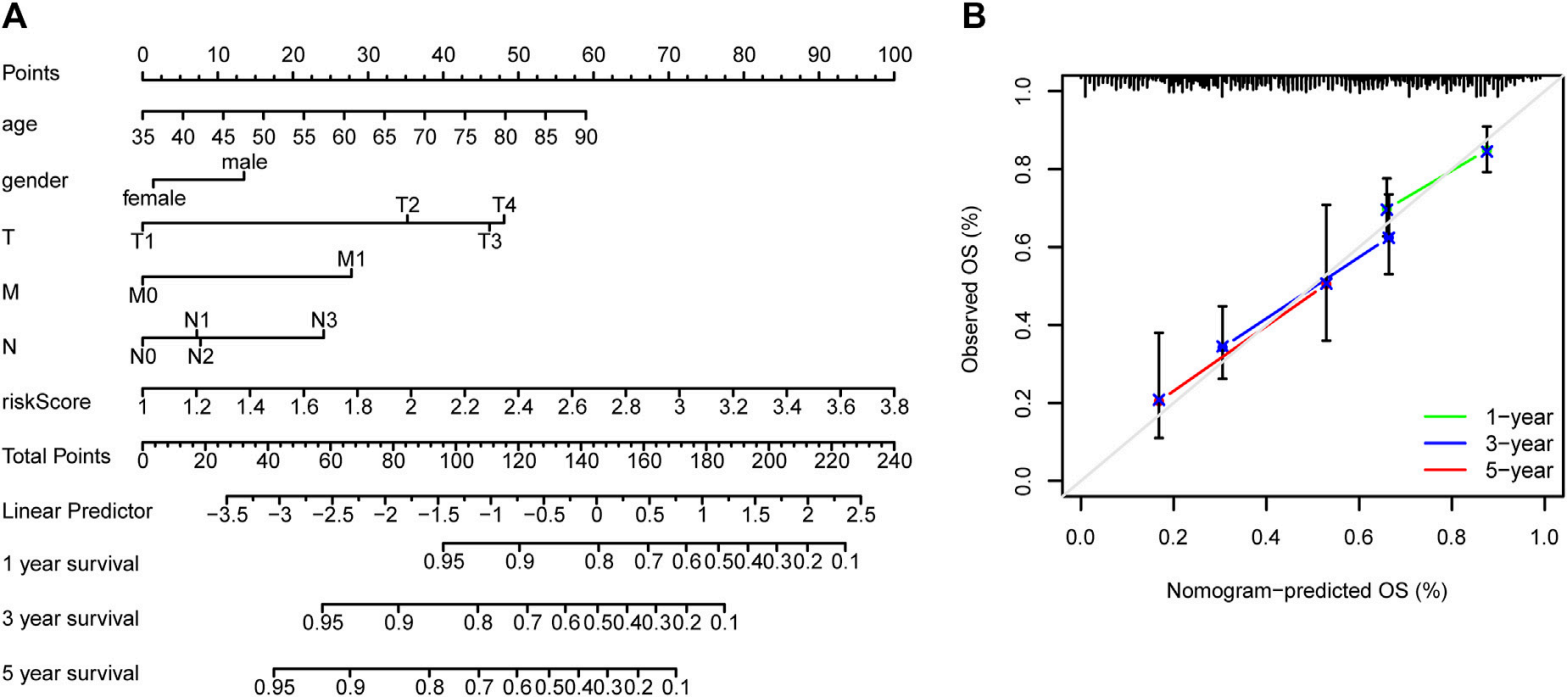
Prediction nomograms. **(A)** Nomogram model based on clinicopathological features and risk scores of patients with gastric cancer in TCGA dataset to predict their prognosis. **(B)** Calibration curve of the nomograms for predicting overall patient survival. The diagonal dotted line represents the ideal nomogram.

TCGA cohort was then divided into low- and high-risk groups based on the median risk score. The Kaplan–Meier curve suggested a lower overall survival rate for patients with GC with high-risk scores than those with low-risk scores (Figure 5C). The area under the curve was more significant than 0.6 for the 1-, 3-, and 5-year survival ROC curves, which indicated that risk scores accurately predicted patient survival rates (Figure 5D). The dataset’s risk score distribution and survival status are shown in Figures 4E,F, respectively. An increase in risk scores was accompanied by an increased risk of patient mortality and a shorter survival time (Figures 5E,F). Given the impact of clinicopathological features (including, age, gender, and TNM staging information) on prognosis, a prognostic nomogram was constructed to predict the survival of patients with GC in TCGA dataset (Figure 6A). The C-index of our nomogram model was 0.683, and the calibration plot (Figure 6B) showed a good fit with actual survival outcomes.

### GSEA

**FIGURE 7:**
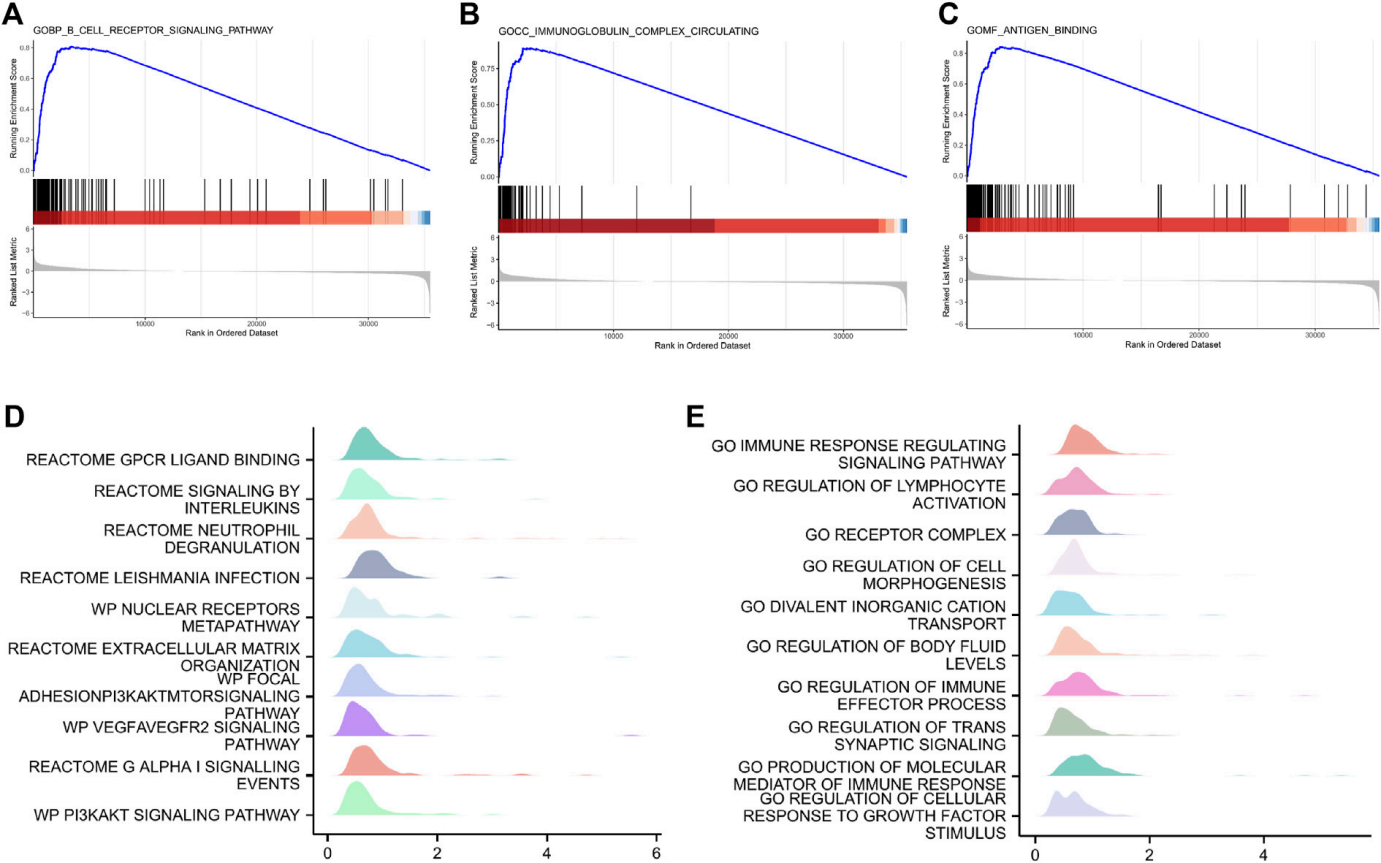
Gene set enrichment analysis showing the nine most important functions or pathways in low- and high-risk patients with gastric cancer in TCGA dataset. **(A)** GOBP: B-cell receptor signaling pathway. **(B)** GOCC: immunoglobulin complex. **(C)** GOMF: antigen binding. **(D,E)** GSEA mountain plot of representative enrichments in C2. all and C5. all MSigDB datasets. Significance was set at *p* < 0.01. GOBP, Gene Ontology biological process; GOCC, Gene Ontology cellular component; GOMF, Gene Ontology molecular function; NES, normalized enrichment scores.

GSEA was performed on all genes to analyze inter-group differences between the low- and high-risk groups. Figure 7 shows the nine most important functions or pathways based on normalized enrichment scores, such as GOBP: B cell receptor signaling pathway, GOCC: immunoglobulin complex circulating, and GOMF: antigen binding. As expected, increased risk scores are substantially associated with high-level immune response.

### Immune cell infiltration

**FIGURE 8:**
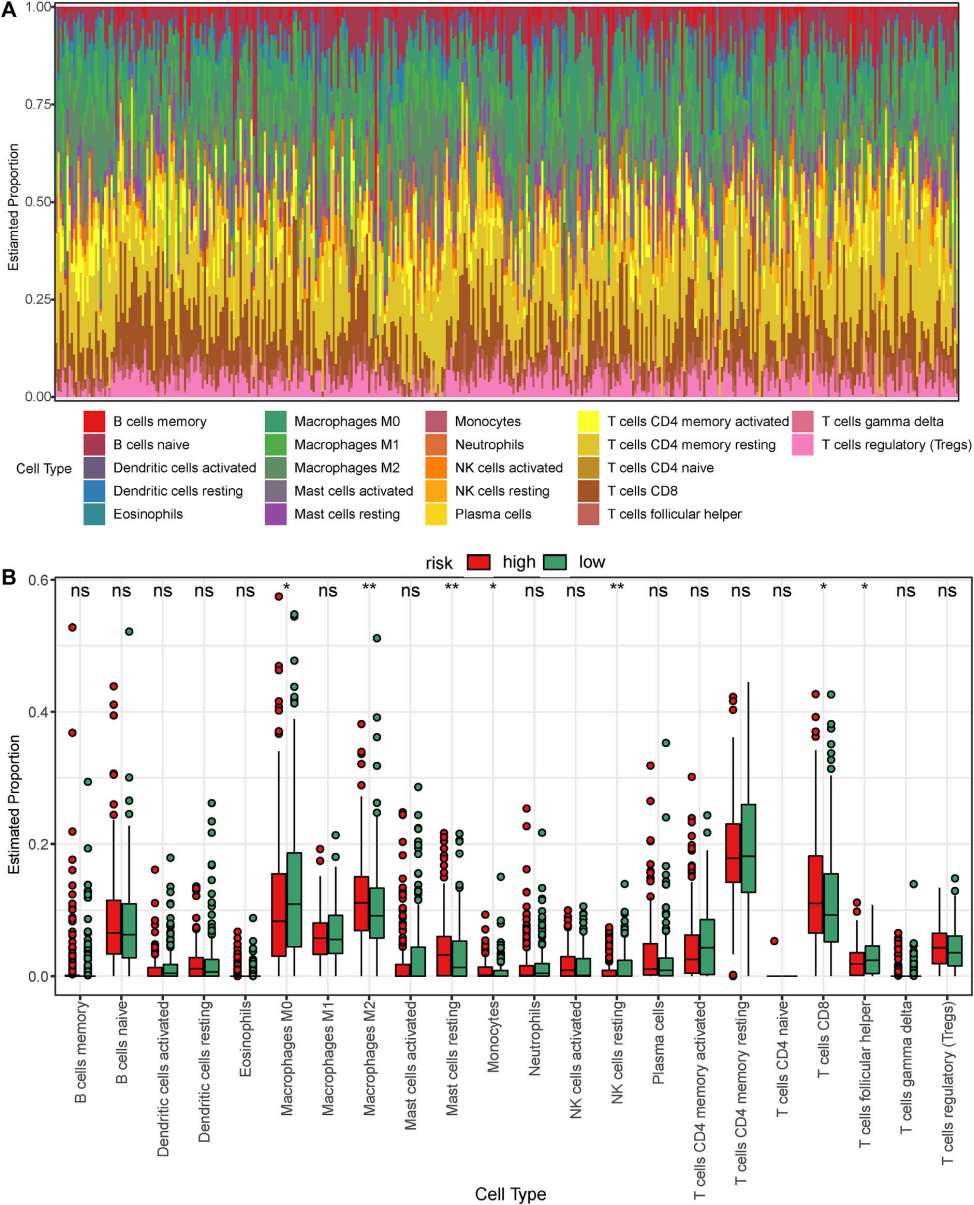
Analysis of immune cell infiltration in the low- and high-risk patients with gastric cancer in TCGA dataset. **(A)** Bar graph showing the proportion of each of the 22 immune cell types in the samples. **(B)** Boxplot comparing the proportion of each of the immune cell types between the low- and high-risk groups. The green and red blocks represent the high- and low-risk groups, respectively.

Based on the previous results, we found that the survival status of the high-risk group was significantly worse than that of the low-risk group and hence speculated that there might exist differences in immune cell infiltration between the low- and the high-risk groups. The CIBERSORT algorithm was used to calculate immune cell infiltration in GC. The bar graph of immune cell infiltration and the boxplot comparing immune cells between the low- and high-risk groups are shown in Figures 8A,B, respectively. We found differences between the two groups in M0 and M2 macrophages, resting mast cells, monocytes, resting NK cells, and CD8 and follicular helper T cells. Interestingly, the expression levels of M0 macrophages, resting NK cells, and follicular helper T cells increased in the low-risk group, which expressed a relatively mild immune response. In contrast, the expression levels of tumor-associated immune cells increased in the high-risk group such as M2 macrophages, resting mast cells, monocytes, and CD8 T cells. Considering that the RNA-seq data of TCGA referred to the expression levels of tissue blocks instead of blood samples, these tumor-immune infiltrates often indicate higher malignancy and worse prognosis rather than protective immunity.

**FIGURE 9:**
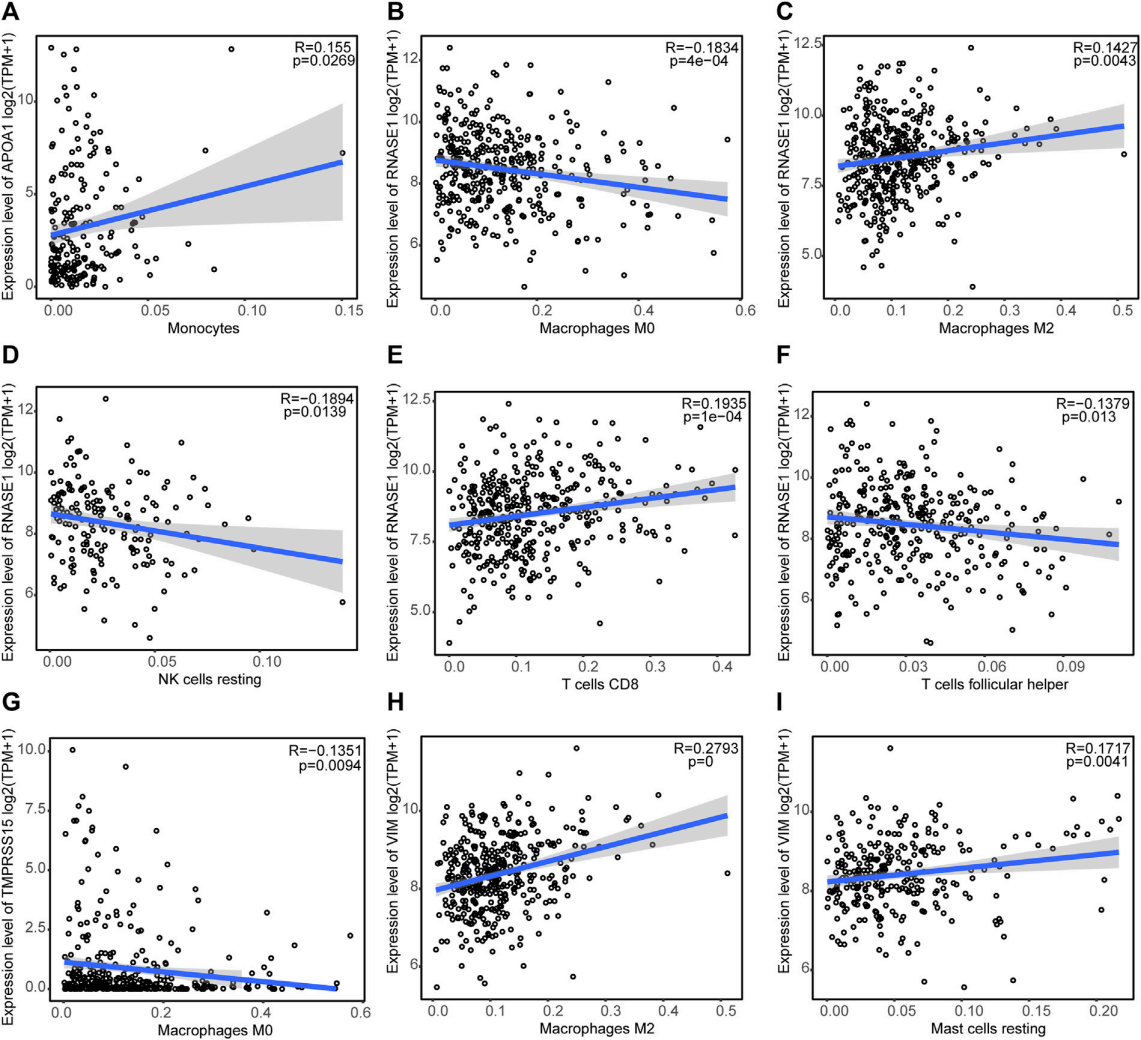
Correlations between the expression of prognostic genes and the levels of immune cell infiltration **(A)** Correlation between *APOA1* expression and the monocyte infiltration level. Correlations between *RNASE1* expression and the infiltration levels of **(B)** M0 macrophages, **(C)** M2 macrophages, **(D)** resting NK cells, **(E)** CD8 T cells, and **(F)** follicular helper T cells. **(G)** Correlation between *TMPRSS15* expression and the M0 macrophage infiltration level. Correlations between *VIM* expression and the infiltration levels of **(H)** M2 macrophages and **(I)** resting mast cells.

Figure 9 shows the correlations between the expression of prognostic genes (*TMPRSS15*, *VIM*, *APOA1*, and *RNASE1*) and immune cell infiltration. *APOA1* expression was positively correlated with the monocyte infiltration level; *RNASE1* expression was positively correlated with the infiltration levels of M2 macrophages and CD8 T cells, and negatively correlated with the infiltration levels of M0 macrophages, resting NK cells, and follicular helper T cells; *TMPRSS15* expression was negatively correlated with the M0 macrophages infiltration level; and *VIM* expression was positively correlated with the infiltration levels of M2 macrophages and resting mast cells, and negatively correlated with the follicular helper T cells infiltration level. This evidence suggested that the prognostic genes for GC are significantly associated with tumor immune infiltration.

## Discussion

Among the numerous advances in the treatment of GC over the last decades, anti-PD-1/PD-L1 immunotherapy has raised particular concerns (Joshi and Badgwell, 2021). TME in GC may influence the response of immunotherapy and affect prognosis (Moutafi and Rimm, 2021). As transcriptomics technologies continue to advance, scRNA-seq offers an excellent tool to research the immune microenvironment biomarkers with clinical translational potential.

The first step toward single-cell analysis is to cluster cell populations with characteristic genes, which is the basis for mapping cell interactions. In addition to the common methods used in this study (Andrews and Hemberg, 2018), a new ensemble random projection-based algorithm, SHARP, which can cluster 10 million cells in large-scale scRNA-seq data, has recently appeared (Wan et al., 2020). CNVs analysis is usually applied for the identification of malignant tumor cells (Patel et al., 2014), but it has unsatisfactory effectiveness in GC (Zhang M. et al., 2021). Our research avoided the controversial topic of separating benign and malignant epithelial cells and instead categorized the component of TME. We found significant differences in the percentage of infiltrating immune cells between early GC and non-cancerous samples and between the low- and high-risk groups in the TCGA. These findings are consistent with a previous comparison of samples before and after cisplatin chemotherapy (Kim et al., 2021). To better clarify the humoral and cellular immune responses in GC, Sathe et al. (2020) concurrently sequenced matched peripheral blood mononuclear cells and revealed the immune remodeling of NK cells, dendritic cells, cytotoxic T cells, and plasma cells. Considering the loss of spatial dimension in cell isolation from tissues, the conjoint analysis of scRNA-seq and spatial transcriptomics in pancreatic cancer (Moncada et al., 2020) and squamous cell carcinoma (Ji et al., 2020) has been proposed, giving us a new perspective on tumor-infiltrating immune cells.

Some differentially expressed non-marker genes confirmed clustering results from another perspective. Overexpression of *GKN1* in epithelial cells (Yoon et al., 2013), *IGLL5* in B cells (Zheng et al., 2021), *TPSAB1* in mast cells (Konnikova et al., 2021), and *RGS5* in smooth muscle cells (Silini et al., 2012) are consistent with previous studies, while *CXCL14* in fibroblasts (Wu et al., 2020) suggests that the conclusion from bulk RNA-seq may not be precise or versatile enough. Together with marker genes, these genes constitute 64 DEGs between IM and EGC, which reflect transcriptional heterogeneity among different cell types. In addition, 9 of 64 DEGs are members of the heat-shock protein family involved in cellular stress responses and pro-tumor inflammation as molecular chaperones. For example, Hsp72 has been previously reported to promote the oxaliplatin resistance of GC cells by inhibiting SDF-2 degradation (Takahashi et al., 2016); Hsp90ab1 is known to facilitate the epithelial–mesenchymal transition in GC *by* preventing LRP5 ubiquitination (Wang et al., 2019). Both genes are highly expressed by enterocytes and can be perceived as indicators of carcinogenicity for extensive intestinal metaplasia.

Macrophage migration inhibitory factor (MIF) is a multipotent cytokine involved in both inflammatory processes and anti-tumor immune response, having the properties of an enzyme, chemokine, and hormone simultaneously (Sumaiya et al., 2021). As a proinflammatory mediator secreted by numerous immune cells, MIF promotes inflammation and autoimmune diseases mainly by binding with the receptor CD74 and co-receptor CD44, CXCR4, or CXCR2 (Liehn et al., 2013; Nakamura et al., 2021; Wallace et al., 2021). Previous studies have shown that the carcinogenic role MIF plays is related to the activation of p53, Ras/MAPK, and Akt pathways in the inflammation–cancer axis (Mittelbronn et al., 2011; Wang et al., 2017; Liu et al., 2018). This study observed the prominent MIF signaling pathway network in GC at the single-cell level. Comparisons and enrichment analysis for DEGs in IM and EGC supported the enriched pathway associated with cell communication and inflammatory response. Mechanistically, MIF-pathway overactivation is likely a manifestation of either the macrophage aggregation caused by *Helicobacter pylori* infection or accumulations of tumor-associated macrophages, which further predisposes epithelial cells to malignant transformation. Under various environmental stimuli, macrophages developing from differentiated monocytes are classified into classically activated (M1) or alternatively activated (M2) macrophages. Generally, IL-12 synthesized and secreted by M1 can induce the proliferation and differentiation of naïve T cells into Th1 cells and enhance the NK cell-mediated antitumor effect; IL-10 secreted by M2 has the opposite effect (Gambardella et al., 2020). Our prognostic model shows that the expression of pro-tumorigenic M2 in the high-risk group is increased, and prognostic genes are also positively correlated with M2 infiltration. A reasonable explanation is that MIF facilitates the M2 polarization of macrophages in GC (Huang et al., 2019; Chen et al., 2021). Recently scholars (Zhou et al., 2022) found that carfilzomib enables M2 to express M1 cytokines, showing great immunotherapeutic potential for solid tumors. For future applications, experimental validation and subgroup analysis of macrophages are required.

Physician-scientists have long grappled with the quantitative assessment of immune TME (Zhang Z. et al., 2021). In advanced GC, Zeng et al. (2021) built a TME scoring system for the effects of checkpoint immunotherapy. By implementing machine learning, Cai et al. (2020) established a prognostic classification model based on the expression of *VCAN*, *CLIP4*, and *MATN3* in a total of 1699 GC patients*.* In our study, four high-risk genes derived from scRNA-seq show the prognostication of poor outcomes. Vimentin (VIM), a type III intermediate filament protein, which characterizes the stromal component of TME in solid cancers, is often regarded as the epithelial–mesenchymal transition marker. It is closely related to GC cell invasion and metastasis, and is the most commonly used marker to detect the acquisition of these mesenchymal traits (Zeng et al., 2018; Zhu et al., 2019). Apolipoprotein A1 (APOA1), a component of high-density lipoprotein, exerts a favorable effect on the prevention of many cardiovascular diseases. However, its elevated level in the urine of bladder cancer patients (Peng et al., 2019) indicated shorter survival. For patients with colorectal cancer, increased APOA1 expression in the blood is concomitant with CD3^+^ T cells aggregation in the core of the tumor as well as the invasive margin (Guo et al., 2019). *TMPRSS15,* known as an enzyme gene, is translated as a serine protease in enterocytes and goblet cells—enteropeptidase. The link between *TMPRSS15* and tumors has rarely been reported before, but other genes coding for the same family proteins were proven to be promising therapeutic targets. *TMPRSS*2 was found to favor the immune escape of COVID-19 (Shang et al., 2020), and *TMPRSS*4 was upregulated in malignancies of the stomach (Tazawa et al., 2022), liver (Dong et al., 2020), and prostate (Lee et al., 2021). In the present study, we speculate that *TMPRSS15* and *APOA1* used for enterocyte identification might be a cellular trait of intestinal type-GC. Therefore, the high expression of these two genes heralded extensive metaplasia and worse outcome. Similarly, despite the little evidence for a direct relationship between RNase1 release in endothelial cells (Bedenbender and Schmeck, 2020) and cancer progression, *RNASE1* shows a good prediction performance based on raw data in TCGA (Li et al., 2020; Yang et al., 2022).

In conclusion, we analyzed the previously published data of scRNA-seq as well as TCGA, explored the inflammation–immunity–cancer axis, and developed a tumor microenvironment-associated risk score in GC. They well reflect the immune infiltration level and help construct a prognostic nomogram model that can assess overall long-term outcomes. Nevertheless, further in-depth studies are needed to confirm our results and broaden our view of TME in GC.

## Data Availability

The original contributions presented in the study are included in the article/Supplementary Material; further inquiries can be directed to the corresponding author.

## Supplementary material

SUPPLEMENTARY FIGURE S1 Analysis of the biopsy specimens from the gastric antral mucosa of patients with non-atrophic gastritis (NAG), chronic atrophic gastritis (CAG), intestinal metaplasia (IM), or early gastric cancer (EGC) based on single-cell RNA-seq data. **(A)** Graphs showing the 48,566 cells included in this study after quality control. **(B,C)** Cluster analysis based on the disease group and cluster grouping. **(D)**Pie chart of cell types. **(E)** tSNE projections of DEGs in different cell types.

SUPPLEMENTARY FIGURE S2 CellChat analyses of the intercellular communication network for different cell types. **(A)** Chord diagram showing the input and output signal pathways in different cell types. **(B)** and **(C)** Number and intensity of cell–cell interaction signal pathways.

SUPPLEMENTARY FIGURE S3 Relative contribution of each ligand–receptor pair in different cell types.

SUPPLEMENTARY FIGURE S4 Complete heat map showing the expression patterns of ten differentially expressed genes (DEGs) between the EGC and IM groups in the different cell types.

SUPPLEMENTARY FIGURE S5 Complete heat map of the correlation between the DEGs.

SUPPLEMENTARY FIGURE S6 Kaplan–Meier (KM) survival curves of four genes in the Kaplan–Meier Plotter.

SUPPLEMENTARY FIGURE S7 Protein expression levels of four genes in HPA. **(A)** Vimentin is highly expressed in stromal cells of GC. **(B)** Enrichment of *APOA1* in tissue-specific cell types. **(C)** *TMPRSS15* is mainly expressed in proximal enterocytes. **(D)** Protein expression of *RNASE1* in the gastrointestinal tract.
