# Supplementary figures and images for: Identification of novel tumor microenvironment-associated genes in gastric cancer based on single-cell RNA-sequencing datasets
Source: Front Genet. 2022 Aug 15;13:896064. doi: 10.3389/fgene.2022.896064 (PMC9421061; doi:10.3389/fgene.2022.896064)

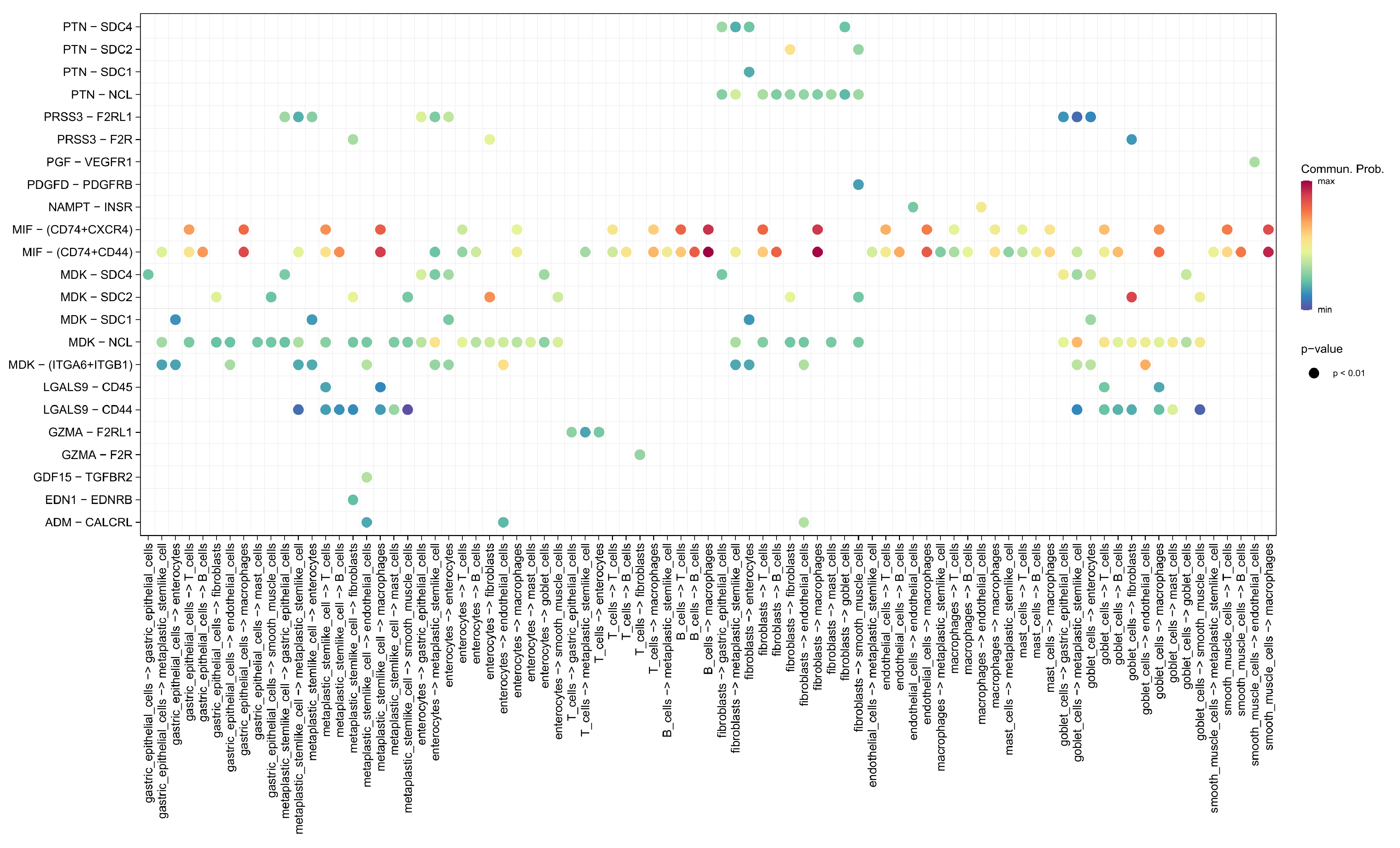

Supplement: Supplementary file 1 [file Image3.TIFF]

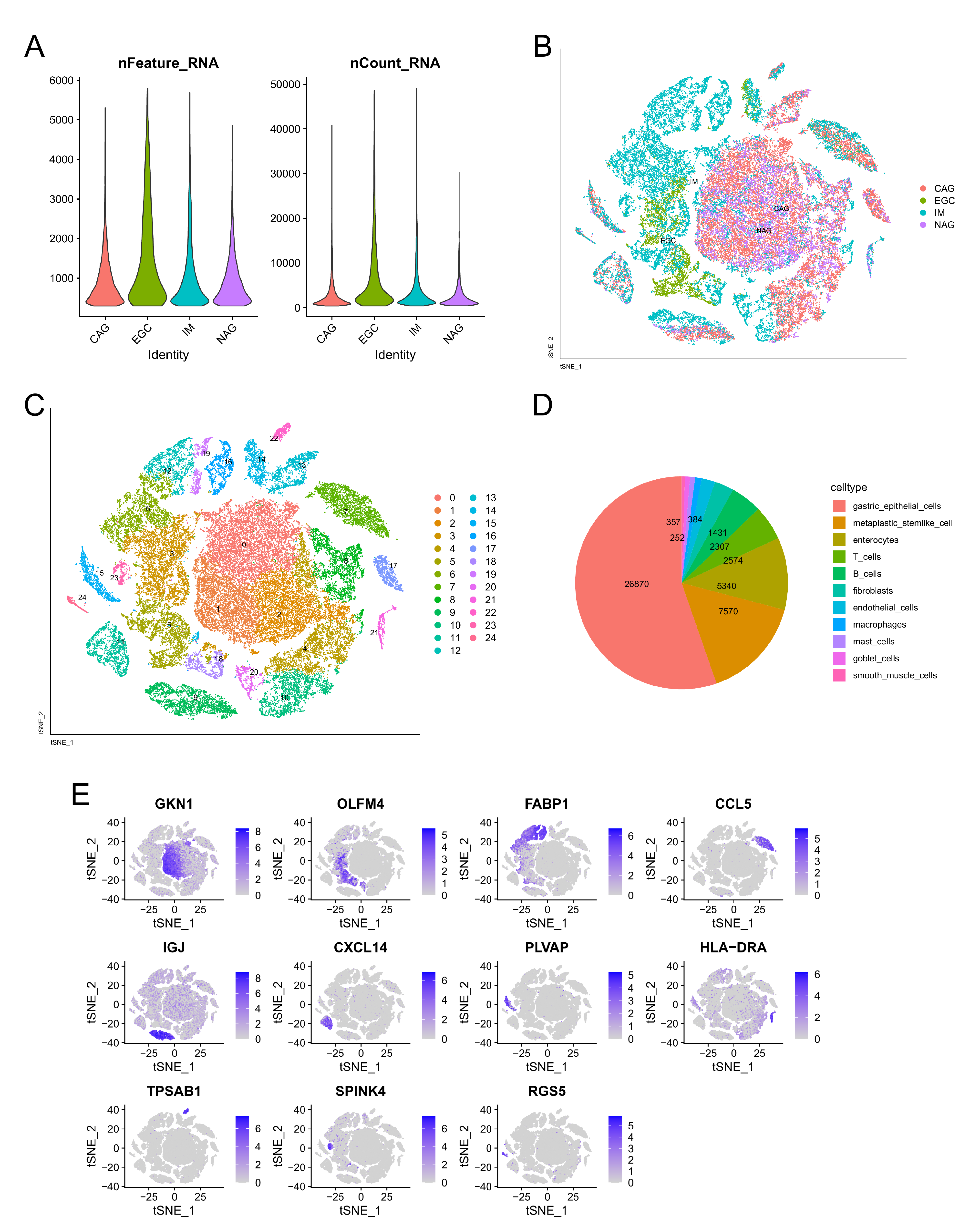

Supplement: Supplementary file 2 [file Image1.TIFF]

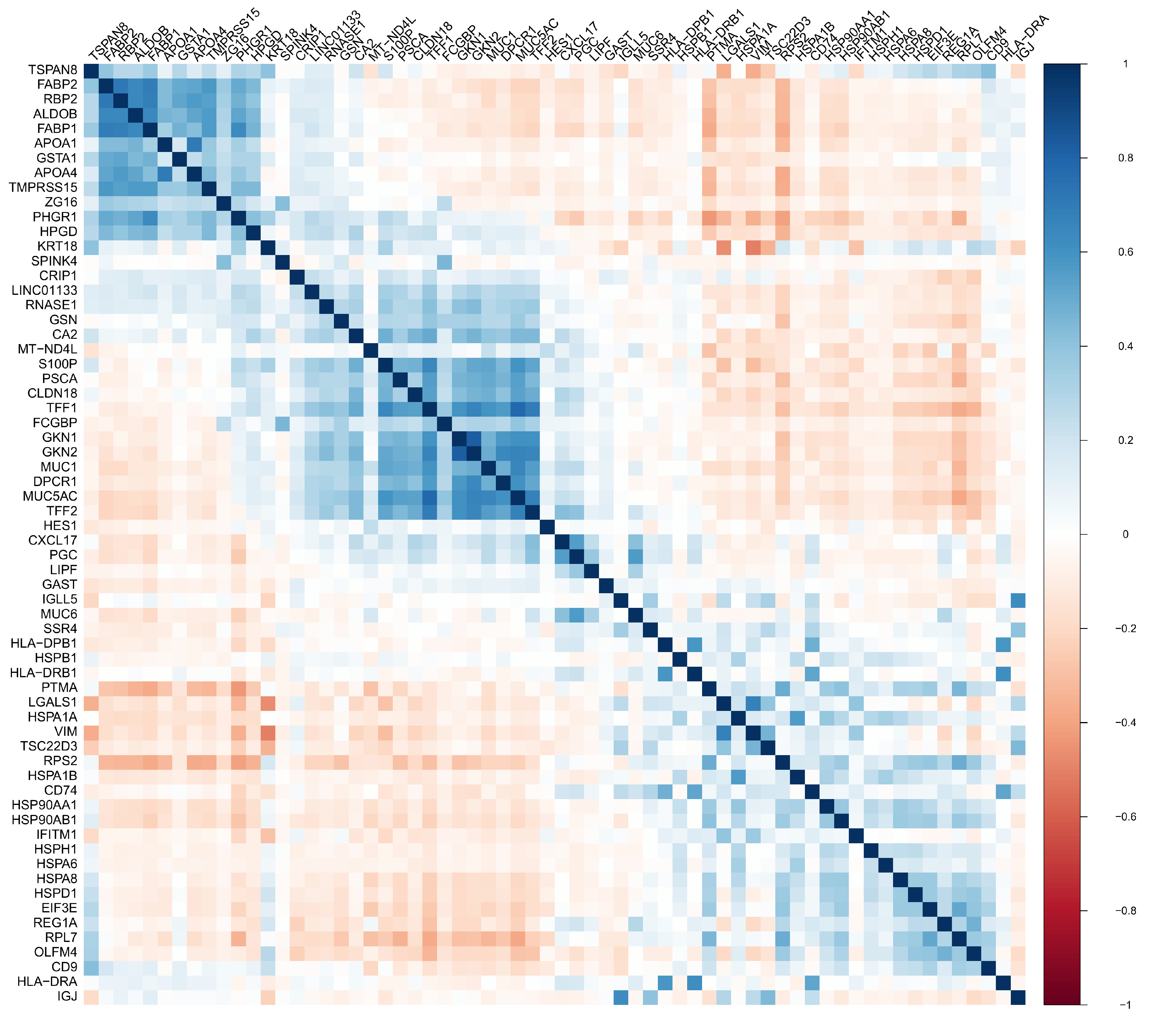

Supplement: Supplementary file 3 [file Image5.TIFF]

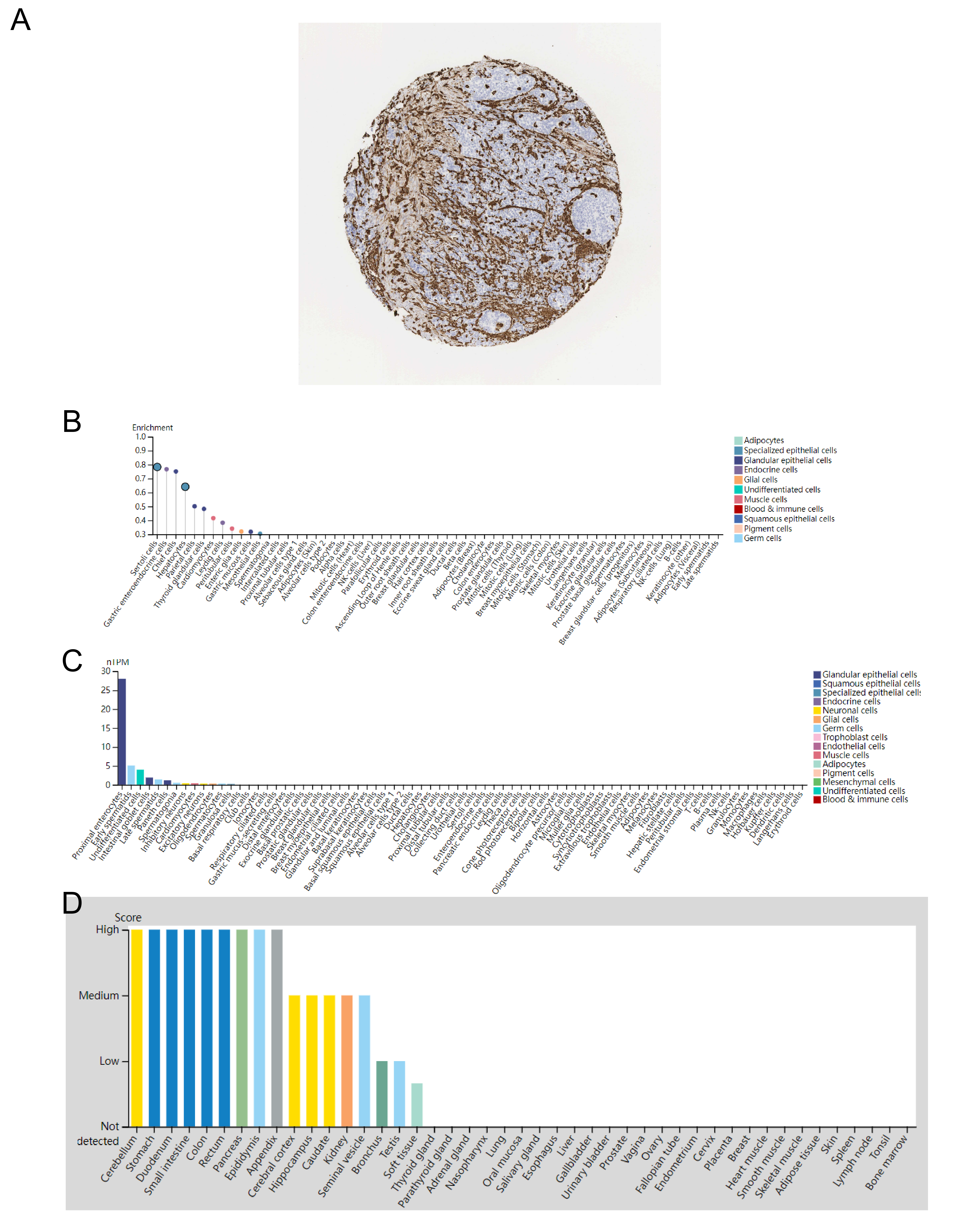

Supplement: Supplementary file 4 [file Image6.TIFF]

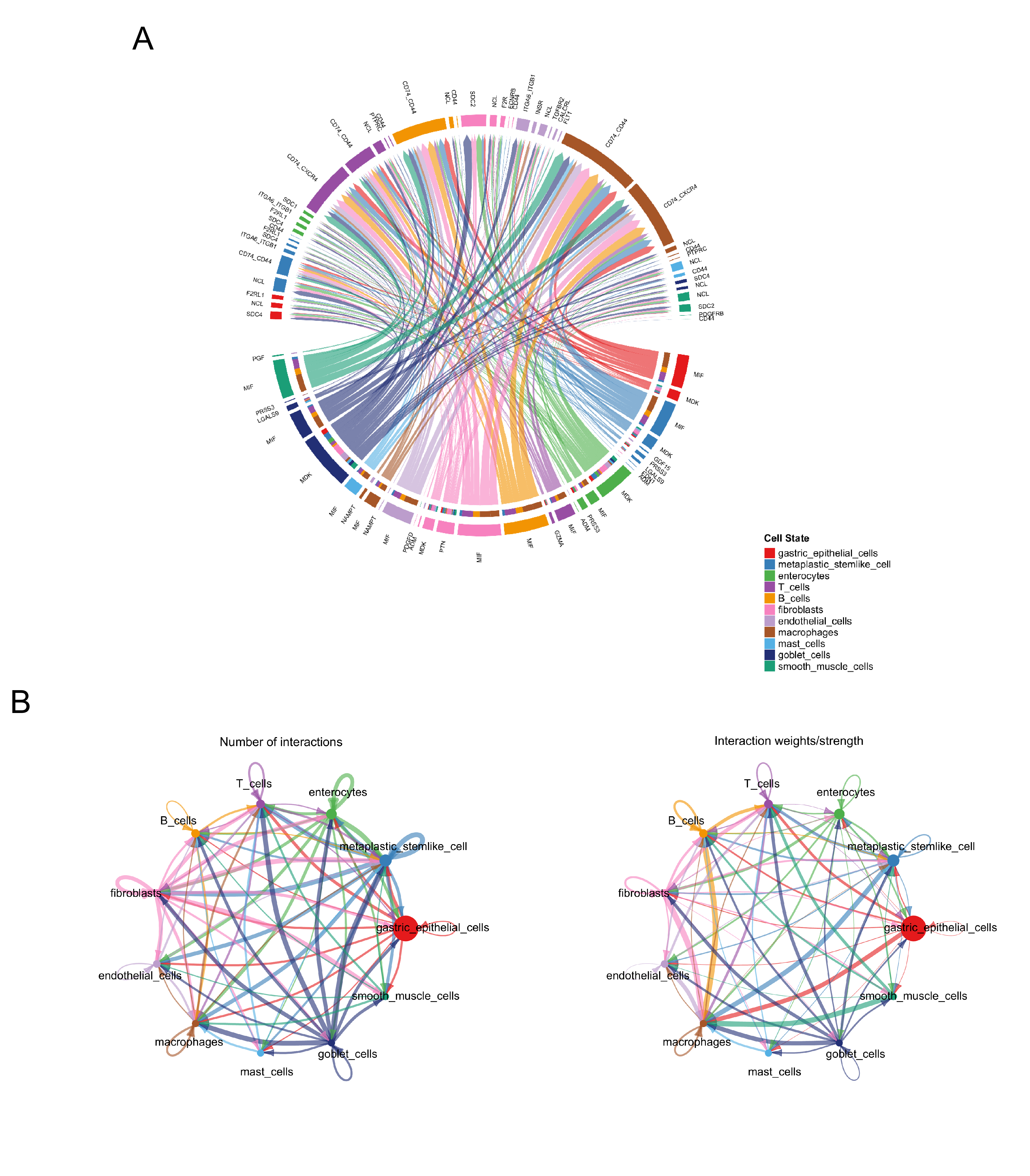

Supplement: Supplementary file 5 [file Image2.TIFF]

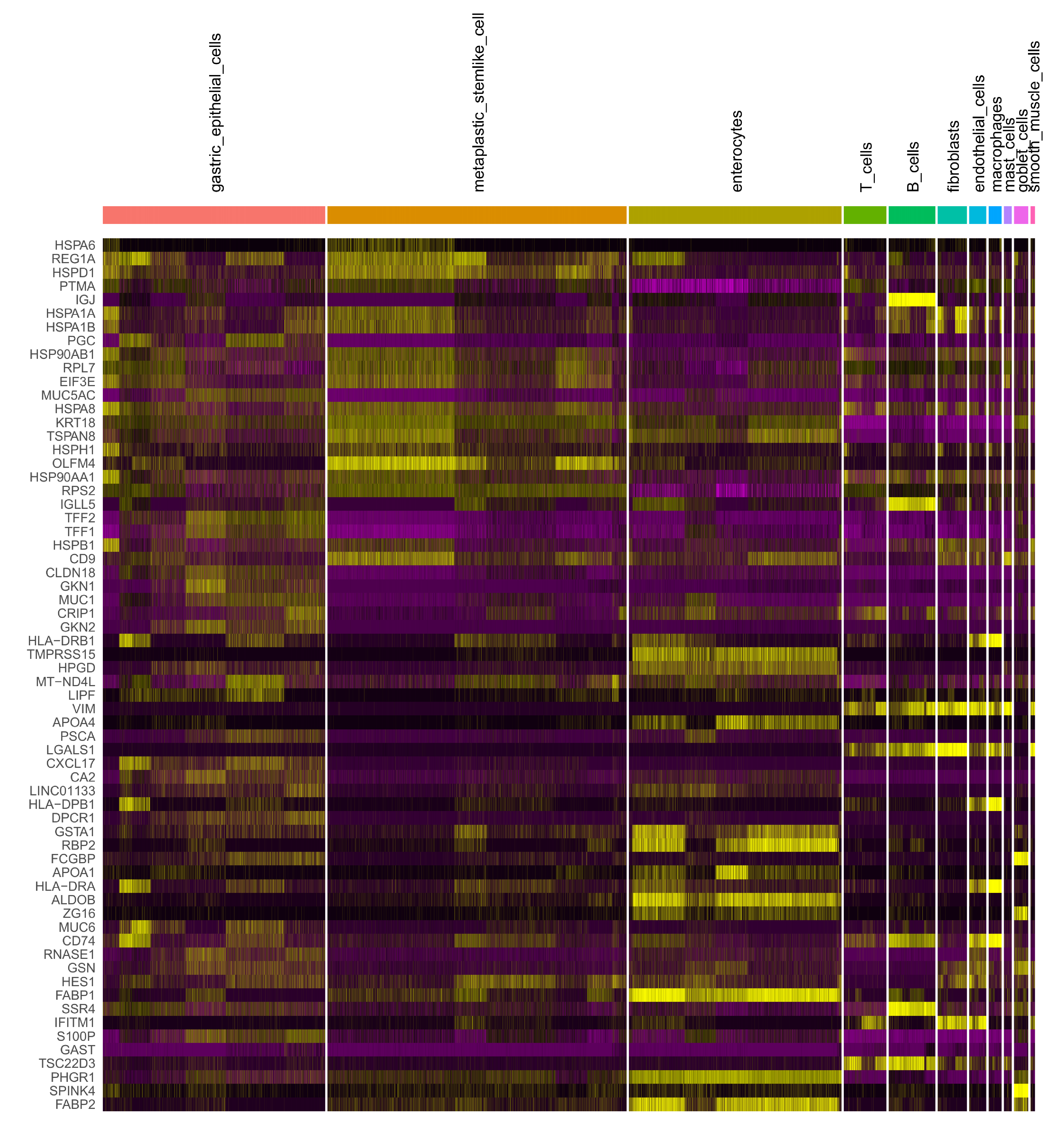

Supplement: Supplementary file 6 [file Image4.TIFF]

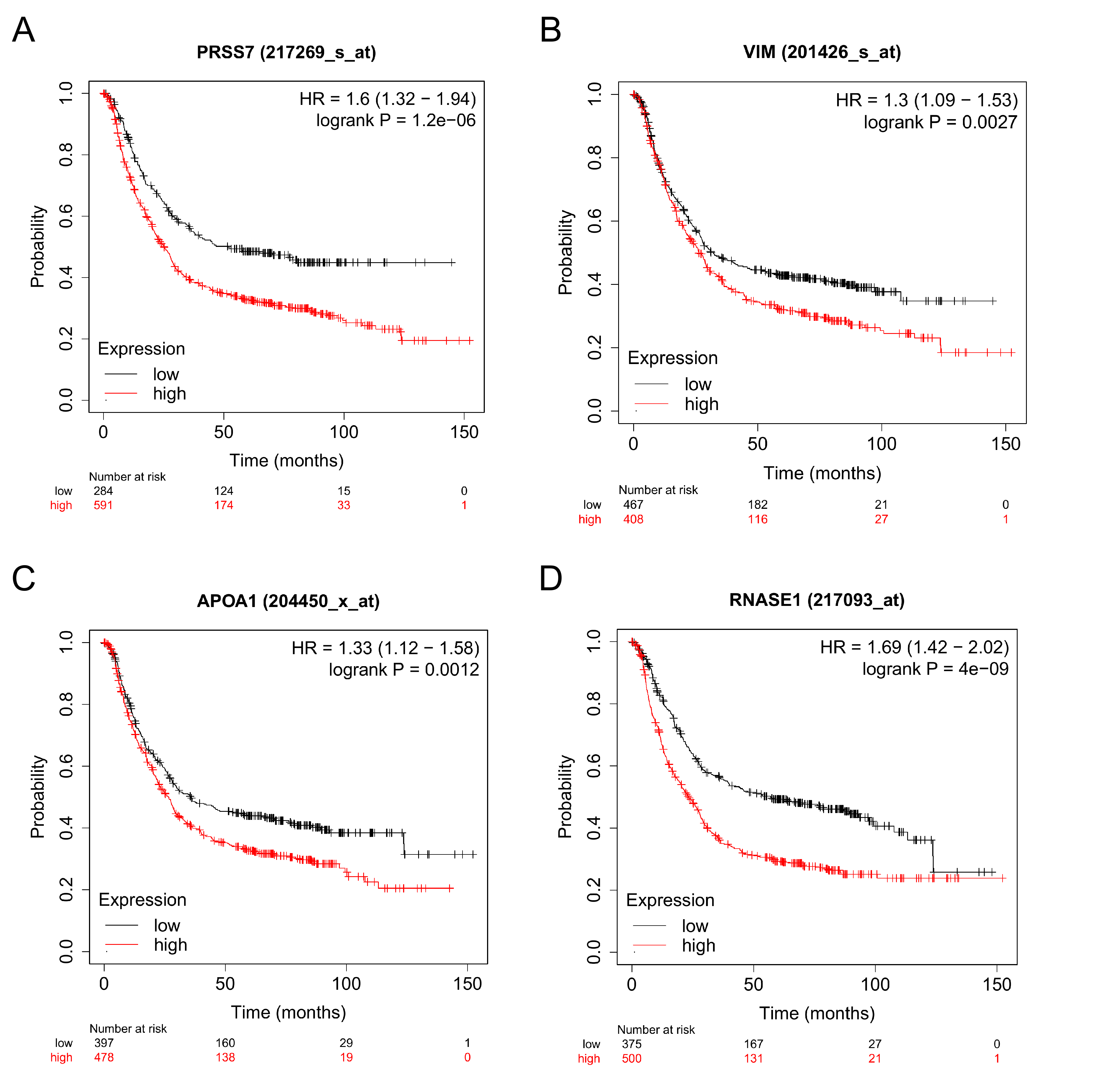

Supplement: Supplementary file 7 [file Image7.TIFF]
